# Multimodal Data Approaches for Examining the 2024-2025 Highly Pathogenic Avian Influenza Outbreak in the United States: Descriptive Study

**DOI:** 10.2196/86209

**Published:** 2026-06-22

**Authors:** Juliana Sopko, Aimee R Han, Jacqueline Powers, Jacquelin A Sauer, Mansi Avunoori, Stanislaw Zakrzewski, Allison Krugman, Abhishek Dasgupta, Kara Sewalk, Autumn Gertz, Benjamin Rader, James Sheldon, Brennan Klein, Jessica Malaty Rivera, Moritz U G Kraemer, Samuel V Scarpino, John S Brownstein

**Affiliations:** 1 Computational Epidemiology Lab, Boston Children's Hospital, 401 Park Drive, 7th Floor West, Boston, MA, 02215, United States, 1 617 355 8278; 2 Technical University of Lodz, Lodz, Poland; 3 Council on Foreign Relations, New York, NY, United States; 4 Department of Biology, University of Oxford, Oxford, United Kingdom; 5 Pandemic Sciences Institute, University of Oxford, Oxford, United Kingdom; 6 Harvard Medical School, Harvard University, Boston, MA, United States; 7 Institute for Experiential AI, Northeastern University, Boston, MA, United States; 8 Network Science Institute, Northeastern University, Boston, MA, United States; 9 Department of Physics, Northeastern University, Boston, MA, United States; 10 Department of Public Health and Health Sciences, Northeastern University, Boston, MA, United States; 11 Vermont Complex Systems Institute, University of Vermont, Burlington, VT, United States; 12 Santa Fe Institute, Santa Fe, NM, United States; 13 Harvard Medical School, University of Ngaoundéré, Boston, Cameroon

**Keywords:** one health, digital health, public health surveillance, zoonotic infectious diseases, zoonotic spillover, avian influenza, wastewater surveillance, data curation, data analysis, data visualization

## Abstract

**Background:**

Highly pathogenic avian influenza (HPAI) A(H5N1) clade 2.3.4.4b, a globally predominant strain, was introduced into poultry in the United States in 2022 via spillover from wild birds, and has since been regularly reported, posing ongoing risks to animal and human health. In 2024, the United States reported the first known HPAI A(H5N1) clade 2.3.4.4b infection in dairy cattle, rapidly evolving into a multispecies outbreak among cattle and poultry, with spillover into humans. Publicly available data remained siloed and fragmented, hindering timely response. Innovative multimodal surveillance methods can enhance situational awareness through comprehensive, standardized data collection, integration, and visualization.

**Objective:**

This study aimed to describe observations from the application of enhanced surveillance methods that collect, integrate, and visualize multimodal data for real-time tracking of the 2024‐2025 HPAI A(H5) outbreak in the United States as an innovative, transparent, repeatable, and scalable approach for open-source public health surveillance.

**Methods:**

Global.health conducted real-time, multimodal surveillance of the United States 2024‐2025 HPAI A(H5) outbreak using publicly available data for human cases (Centers for Disease Control and Prevention), animal outbreaks (United States Department of Agriculture), wastewater monitoring (WastewaterSCAN), genomic data (public genomic databases), research updates (scholarly communication), and policy changes and response measures (media and government) for the study period from February 1, 2024 through February 28, 2025. This digital data stream was used to create outbreak resources—an epidemiological linelist, event timeline, and interactive map—using a One Health framework to track emerging hotspots.

**Results:**

Global.health curated 70 confirmed human HPAI A(H5) cases across 13 states in a linelist, with exposure for nearly all (n = 65, 92.9%) cases associated with commercial agriculture and related operations. We curated 682 timeline entries across 6 distinct categories: human, cattle, response (eg, research, policy changes, and public health guidance), birds, genome, wastewater, and mammals. The map integrated human cases (n=70) and animal outbreaks (commercial cattle: n=977 and commercial poultry: n=325) into a single view. California was identified as the outbreak epicenter with high numbers of human cases (n=38, 54.3%), commercial cattle outbreaks (n=748, 76.6%), and commercial poultry outbreaks (n=66, 20.3%) during the study period. Wastewater surveillance detected the virus in California, with an unknown source at least 81 days before the first confirmed commercial dairy cattle case.

**Conclusions:**

Global.health’s approach for integrating traditional and nontraditional public health surveillance data within a One Health framework enhanced early situational awareness during the United States 2024‐2025 HPAI A(H5) outbreak, creating open access to resources that improve contextual understanding of the scope and evolution of this emerging zoonotic event. Further research should seek to understand the full potential of multimodal data in outbreak surveillance.

## Introduction

### Background

The global spread of the highly pathogenic avian influenza (HPAI) virus (A/goose/Guangdong/96-lineage virus of the A(H5) subtype) has resulted in many different clades and genotypes due to genetic mutations, drift, and reassortment [1,2]. The H5Nx goose/Guangdong lineage in clade 2.3.4.4, circulating since 2013, has evolved into 8 different clade variants (2.3.4.4a-h), with clade 2.3.4.4b emerging as a notably fit virus with expanding geographic and host range and becoming widespread globally [3-5]. In early 2022, HPAI A(H5) clade 2.3.4.4b virus was detected in the United States poultry via spillover from wild birds and has since been regularly reported on US poultry farms, continuing to pose a threat to human and animal health [1,6-8].

Early in 2024, farmers in the United States began to report the emergence of a mysterious illness among their cattle herds. This cluster of symptoms was later linked to HPAI A(H5N1) clade 2.3.4.4b virus infection [9]. HPAI is better known for infecting wild and domesticated bird populations, with occasional spillovers into mammals and humans [10]. This appears to be the first time that HPAI (H5N1) has been identified in dairy cattle, aside from controlled infection experiments conducted in 2006 by the Centers for Disease Control and Prevention (CDC) [11-15]. On April 1, 2024, the Texas Department of State Health Services announced the first human case of HPAI A(H5N1). This was the first human case identified since the country’s first and only previous confirmed human case of HPAI A(H5N1) in 2022 [16-18]. The individual was a dairy farm worker who had come in contact with infected cattle while at work. Genomic testing of the virus provided strong evidence that the farm worker had been infected via a spillover from dairy cattle and is believed to be the first mammal-to-human transmission of the HPAI H5N1 virus [14,17]. This spillover into humans marked the start of a multistate zoonotic outbreak affecting humans, cattle, poultry, and other mammals [19].

The emergence of a public health threat, such as a zoonotic outbreak, initiates a series of mitigation efforts. Within the United States, multiple local, state, and federal agencies are responsible for protecting and monitoring human, environmental, and animal health [20-22]. This often results in a complex and fragmented response and surveillance system for monitoring multispecies diseases such as HPAI. A key challenge arising from this surveillance landscape is that data collected by responding agencies are often siloed, which in turn can hamper timely analysis and response [23]. Barriers to implementing One Health surveillance systems—including data privacy, quality, and resource capacity—mirror longstanding challenges in the evolution of public health surveillance, highlighting the need for innovative methods to improve monitoring of zoonotic diseases [24,25].

Global.health was formed during the early stages of the SARS-CoV-2 pandemic in 2020 to address the challenge of integrating fragmented data during multiagency, multijurisdictional outbreak responses [23,26-28]. The technology powering Global.health is an open-source, scalable, and accessible platform for the rapid curation, analysis, and visualization of early outbreak data using a common data language for standardized database entry at a global scale [29]. The platform has been deployed in response to mpox, Ebola, and Marburg outbreaks, evolving to address the specific needs presented by each event [30-32]. Metadata associated with individual epidemiological cases can be of great importance to understand early disease dynamics, including origins, transmission dynamics, and key epidemiological parameters.

### Study Objectives

To increase access to data and support One Health analysis of the 2024‐2025 HPAI A(H5) outbreak in the United States, Global.health launched a data collection effort integrating human cases, animal outbreaks, wastewater surveillance, genomic data, research, response efforts, and policy actions. Accompanying this effort was the creation of enhanced data visualizations to improve outbreak insights. In this study, we examine Global.health’s multimodal data collection, integration, and visualization efforts. Subsequently, we discuss observations regarding the spread of HPAI A(H5) in the United States during 2024 and 2025. Using information collected between February 2024 and February 2025, this paper highlights the value of integrating disparate datasets into infectious disease surveillance systems to improve situational awareness.

## Methods

### Outbreak Initiation

**Figure 1.**
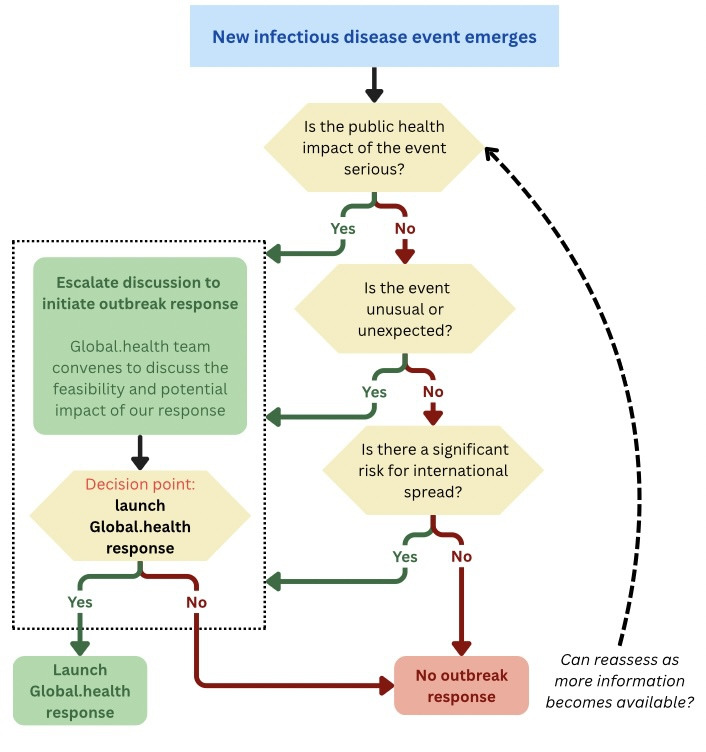
Global.health decision instrument for the assessment of an emerging infectious disease event.

A set of established protocols are used to analyze emerging public health events and determine if they require the launch of Global.health outbreak response [29]. Early in 2024, the Global.health team initiated an outbreak response to the 2024‐2025 HPAI A(H5) outbreak in the United States. The launch of an outbreak response triggers a cascade of planning and preparatory measures outlined in Figures1  2.

**Figure 2.**
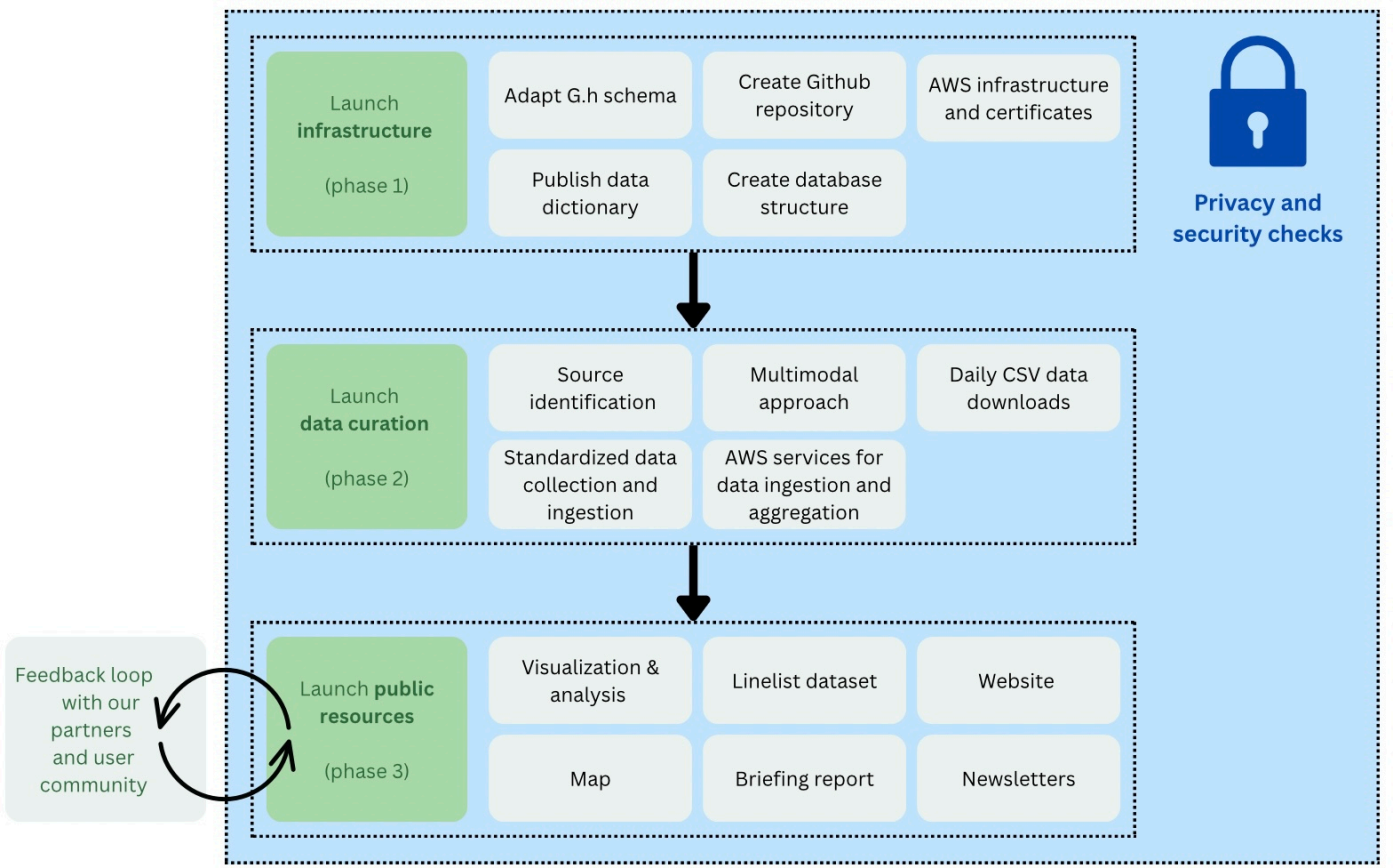
Outbreak response workflow. AWS: Amazon Web Services; CSV: comma-separated values.

Figure 1 modeled after the World Health Organization’s International Health Regulations (2005) Third Edition Annex 2, a cascade of yes or no binary questions are used to support informed decision-making [33]. A “yes” response to these questions will escalate internal discussion around the feasibility and potential impact of our work, considering timing, team resources, publicly available information, and current event coverage. This assessment is used to determine whether or not a new infectious disease event warrants the activation of a Global.health outbreak response. The outcome of this discussion reaches a decision point (yes or no) to launch the Global.health outbreak response. A “yes” decision will move forward with the launch of a Global.health outbreak response outlined in Figure 2. A “no” decision can be reassessed at any time as more information becomes available. Serious public health events include those with high case-fatality rates, limited access to effective prophylaxis or treatment, challenges in detecting the pathogen, spread in community and health care settings, and the need for an enhanced response at multiple levels. A subset of pathogens that Global.health considers as likely triggers for serious public health events are listed by the United Kingdom Health Security Agency as high-consequence infectious diseases [34]. Global.health may respond to other high-consequence infectious diseases such as mpox and measles, as well as vector-borne diseases such as dengue, chikungunya, and yellow fever.

Decision to launch a Global.health response triggers a triphased series of actions that first sets up the infrastructure (phase 1), then launches data curation efforts (phase 2), and later provides public resources to our global user community (phase 3). Examples of the types of activities or products created to support each phase are listed in the relevant section. A feedback loop is built into the model to support a transparent, crowdsourced approach to sharing outbreak information and to enable meaningful collaboration and fact checks through our platform. We consider relevant data privacy laws and regulations and use robust data protection measures and mitigation strategies, including deidentification, data validation, data quality checks, and access control.

### Data Collection: Outbreak Linelist, Human Data

Global.health developed and maintains a common data schema used to structure infectious disease data. During a response, the Global.health outbreak schema is applied and may be adapted for novel data streams or pathogens to create the outbreak linelist that records granular epidemiological data pertaining to human cases in a standardized manner, building on lessons learned from COVID-19 data curation [35,36]. The outbreak schema is used for every outbreak (Multimedia Appendix 1) and can be modified to add new fields of interest. The schema format standardizes key epidemiological outbreak data that are deidentified. The availability of each data element varies by pathogen, location, and source, among other factors. For the 2024‐2025 HPAI A(H5) outbreak, the Pathogen_name field was expanded to capture subtype detail for HPAI; Pathogen_subtype1 and Pathogen_subtype2 capture data for hemagglutinin and neuraminidase proteins, respectively. The genomics field was expanded to capture distinct information for sequence, clade, and genotype when that information was publicly available. The study period captures and reviews outbreak data in the United States between February 1, 2024, and February 28, 2025. Systematic data collection started in April 2024 and retrospectively added earlier events of significance. Research and data collection were conducted using secondary data from publicly available, deidentified sources and did not involve human participants. This study followed the STROBE (Strengthening the Reporting of Observational Studies in Epidemiology) reporting guideline. Data collection is conducted by a team of curators who undergo extensive training for data collection and standardization. Curators accessed the same publicly available data directly from source websites. No restricted databases were used. Each entry is reviewed and validated by at least one additional curator before posting. Data cleaning included standardization of variable names, removal of duplicate entries, and reconciliation of conflicting reports across sources. No imputation of missing data was performed.

The outbreak linelist includes both confirmed and probable human cases of HPAI in the United States, but only confirmed cases are analyzed in this study. Confirmed cases are defined by the Council of State and Territorial Epidemiologists as those that have tested positive for H5 at a public health laboratory and have been confirmed by testing at the CDC [19,37]. Global.health’s primary source for human case data was the CDC in addition to reports from local or state health departments.

Exposure source information reflects what was publicly reported by the CDC and was not defined by the researchers. The exposure source can be one of four categories: dairy herds, poultry farms and culling operations, other animal exposure, and exposure source unknown [19]. For dairy herds and poultry farms, the exposure was associated with commercial agriculture and related operations [19]. For other animal exposure, the exposure was related to other animals such as backyard flocks, wild birds, or other mammals [19]. When the exposure source is unknown, it means the exposure source was unable to be identified following public health investigations [19].

### Data Collection: Outbreak Timeline, Multimodal Data

**Figure 3.**
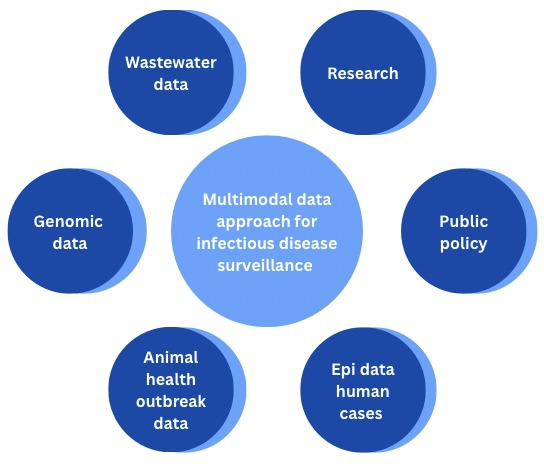
A multimodal data approach for integrated infectious disease surveillance.

Complementing the outbreak linelist and to help place the events in context, an outbreak timeline was created [38]. Because the outbreak linelist is exclusive to human cases, an outbreak timeline was developed that used multimodal data to enhance outbreak surveillance methods and weaved together publicly available, diverse data sources (outlined in Figure 3) into a single, integrated chronology that adopted a One Health approach, highlighting the interconnectedness of humans, animals, and our shared environment for this zoonotic outbreak [39]. The outbreak timeline includes entries related to the 2024‐2025 HPAI A(H5) outbreak during the study period, February 1, 2024 through February 28, 2025. Systematic data collection started in April 2024 and retrospectively added earlier events of significance. Data collection for this event also extended beyond the study period but is not evaluated in this study.

This figure outlines the types of sources collected to track the emerging H5 HPAI outbreak in the United States. Disparate data sources included human epidemiologic data, commercial cattle and poultry outbreak reports, wastewater detections, genomic sequences, research updates, response measures, and policy actions. The integrated view supports early situational awareness for zoonotic events.

Outbreak timeline entries were organized under four variables: date, event, read more (source link), and category. Each entry was tagged under one of the following categories for easy filtering and organization: human, cattle, response, birds, wastewater, genome, and mammals.

The “human” category included information related to HPAI infections in humans, including case counts and research updates. Our primary sources for human data were press releases and reports from the CDC and local and state health departments.

The primary source used to identify animal outbreaks was the United States Department of Agriculture (USDA) [40]. The USDA website reports HPAI in commercial and backyard flocks, wild birds, mammals, and livestock. For poultry outbreaks included in the “birds” category, using the USDA website, curators were able to ascertain the confirmation date, state and county location, production type, control area release, and the number of birds affected [8]. Only poultry or birds classified under commercial production, including the World Organization for Animal Health (WOAH) poultry category, were included in the descriptive analyses for this study. For livestock outbreaks in the “cattle” category, curators used the USDA website to ascertain confirmation date, state location, production type, and species [41]. Outbreaks among “cattle” species and “dairy milking cows” as the production type were included in these analyses. The “mammals” category provides updates on HPAI in other mammals besides cattle and humans.

For the collection of wastewater data, curators manually reviewed publicly available WastewaterSCAN reports from 192 wastewater testing sites across the United States [42]. WastewaterSCAN is an academic partner of the CDC’s National Wastewater Surveillance System (NWSS), and system data are collected and reported to the CDC [43]. WastewaterSCAN provided early, open-access to routinely tested wastewater surveillance data for the H5 subtype with results displayed on a public dashboard, updated daily, starting in May 2024 [42,44]. Filters were selected for pathogen (influenza), view by location, subtype (H5), locations (national, all wastewater sites, n=192). Data are available from WastewaterSCAN as of May 7, 2024, and are available for the remainder of the study period. Positive detections should be interpreted with consideration of the complexities of H5 in wastewater, primarily noting the inability to distinguish between human and animal sources (both domesticated and wild), as discussed in this research.

For the collection of genomic data, including clade and genotype information, sequence name, and accession number, the curation team gathered details from research and technical updates and information submitted to the Global Initiative on Sharing All Influenza Data virus genomic database or to GenBank, the genetic sequence database run by the US National Institutes of Health [45,46]. Data collection and reconciliation efforts for HPAI are ongoing.

The “response” category highlights response efforts, including policy mandates and changes to recommendations and guidelines as the outbreak evolved. This category also encompasses new research related to the response to the outbreak, such as the development of diagnostics and animal vaccines. Sources for “response” entries include news outlets, government agencies, preprint servers, and research journals.

**Figure 4.**
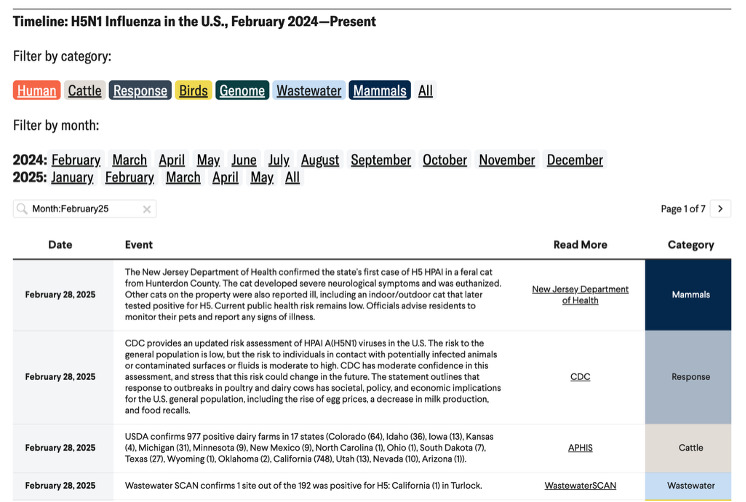
Outbreak timeline.

Curators performed daily source checks, and all timeline entries were reviewed by at least one additional curator before posting to help ensure accuracy and consistency in reporting. Global.health collaborated with Think Global Health to publish a weekly version of the outbreak timeline (Figure 4) [38].

### Visualizing Multimodal Data

The outbreak visualization map was created to accompany the outbreak linelist and multimodal outbreak timeline as publicly available resources [47]. Based on our development experience with maps from COVID-19, mpox (2022 and 2024), Ebola (Uganda 2022), and Marburg (Equatorial Guinea 2023), we used the Mapbox Boundaries and Mapbox GL JS library to create a state-level human HPAI map and later added new data overlays for county-level animal outbreaks in commercial cattle and poultry. Further visualization methods are described in Multimedia Appendix 2 [48].

### Ethical Considerations

No institutional review board approval was applied for. Research was conducted using secondary data from publicly available sources that are deidentified. The research monitored public health signals without involving human participants and was designed to provide timely situational awareness and contribute to generalizable knowledge.

## Results

### Outbreak Linelist, Human Data

As of February 28, 2025, there were a total of 70 confirmed human cases of HPAI A(H5N1) reported in 13 states across the United States [5,19]. Only one confirmed human case of HPAI A(H5N1) had ever been documented in the United States before this outbreak, underscoring a sharp rise in incidence [10,17,49]. Included cases were publicly reported (eg, confirmed human HPAI cases meeting source-defined criteria for our setting and study period). Data availability varied across sources. due to public reporting practices; no data linkage was performed. Nearly all confirmed human cases (92.9%) were from exposure associated with commercial agriculture and related operations. Among the confirmed human cases, 58.6% (n=41) occurred after exposure to infected commercial dairy herds. Additionally, 34.3% (n=24) resulted from exposure to infected commercial poultry farms and culling operations. Two human cases were exposed to other animals (such as backyard flocks, wild birds, or other mammals). For 3 cases, 1 Missouri resident and 2 Californian children, the source of exposure was unknown [19].

H5N1 was the only pathogen subtype that had been identified among these human cases. The sequencing of specimens showed that clade 2.3.4.4b of H5N1 was responsible for these infections, which was the same clade reportedly causing the outbreaks in cattle, poultry, and wild birds [5,50-52]. During the study period, of these sequenced human specimens where data were publicly available, 3 genotypes of clade 2.3.4.4b had been identified: B3.13, D1.1, and D1.3 [10,52,53]. There was no evidence of human-to-human transmission of the virus as of February 28, 2025 [19]. All confirmed human cases during the study period with a known exposure source had resulted from zoonotic, animal-to-human transmission. As of February 28, 2025, there had been only one death reported within the United States [19]. The case fatality rate for the US outbreak was 1.4%. Sixty-one confirmed human cases reported some kind of symptom associated with HPAI as of February 28, 2025. When specific symptoms were noted (Table 1), the 3 most common symptoms reported were fever, conjunctivitis or eye discomfort or watery discharge, and cough. Global.health findings align with those reported in publications in the *New England Journal of Medicine* and *Nature Medicine*, whose researchers analyzed the symptomatology of individuals with confirmed HPAI infections [54,55].

**Table 1.** Symptom variables and their respective definitions used in our clinical analysis of confirmed human HPAI cases. Definitions include alternate terms for variables, which were grouped to avoid duplicates. These variables were extracted from the human case line list dataset. These symptoms were reported among the 61 individuals with confirmed HPAI infections in the United States between February 1, 2024, and February 28, 2025.

Variable	Definition
pink_eye_conjunctivitis	“pink eye” “conjunctivitis” “eye discomfort” “eye redness”
watery_eyes	“watery discharge” “eye tearing” “drainage” “discharge”
subconjunctival_hemorrhage	“subconjunctival hemorrhage”
upper_respiratory_tract	“upper respiratory tract symptoms” “mild upper respiratory symptoms”
respiratory_symptoms	“common respiratory infection symptoms” “severe respiratory illness” “respiratory symptoms”
sore_throat	“sore throat”
runny_nose	“runny nose”
chest_pain	“chest pain”
nausea	“nausea”
weakness	“weakness”
vomiting	“vomiting”
diarrhea	“diarrhea”
nasal_congestion	“nasal congestion”
fatigue	“fatigue”
fever	“fever”
cough	“cough”

Table 1 outlines the symptom variables and their respective definitions used in our clinical analysis of confirmed human HPAI cases. Definitions include alternate terms for variables, which were grouped to avoid duplicates. These variables were extracted from the human case linelist dataset. These symptoms were reported among the 61 individuals with confirmed HPAI infections in the United States between February 1, 2024, and February 28, 2025.

### Outbreak Timeline, Multimodal Data

Early in the outbreak, the Global.health team partnered with Think Global Health, a digital publication by the Council on Foreign Relations supported by Bloomberg Philanthropies. Think Global Health is an online bulletin that examines the ways in which changes in health are reshaping economies, societies, and the everyday lives of people around the globe. Think Global Health published data curated by the Global.health team, creating the outbreak timeline (Figure 4) [38]. During the study period, the outbreak timeline was the 16th most viewed story on the Think Global Health website and recorded over 5800 page views from its publication through February 28, 2025. It also experienced periodic surges in traffic such as when it was first published in June 2024, with additional spikes in October 2024 and January 2025. The outbreak Timeline is accessible directly through the Global.health website, GitHub, and ThinkGlobalHealth.org [26,38,56].

Figure 4 outlines the outbreak timeline published on the Think Global Health website, which presents events related to HPAI in the United States between February 1, 2024, and February 28, 2024 [38]. The timeline allows readers to sort entries by category or month. Each entry provides the date, a brief description of the event, a source link for more details, and the assigned category.

**Figure 5.**
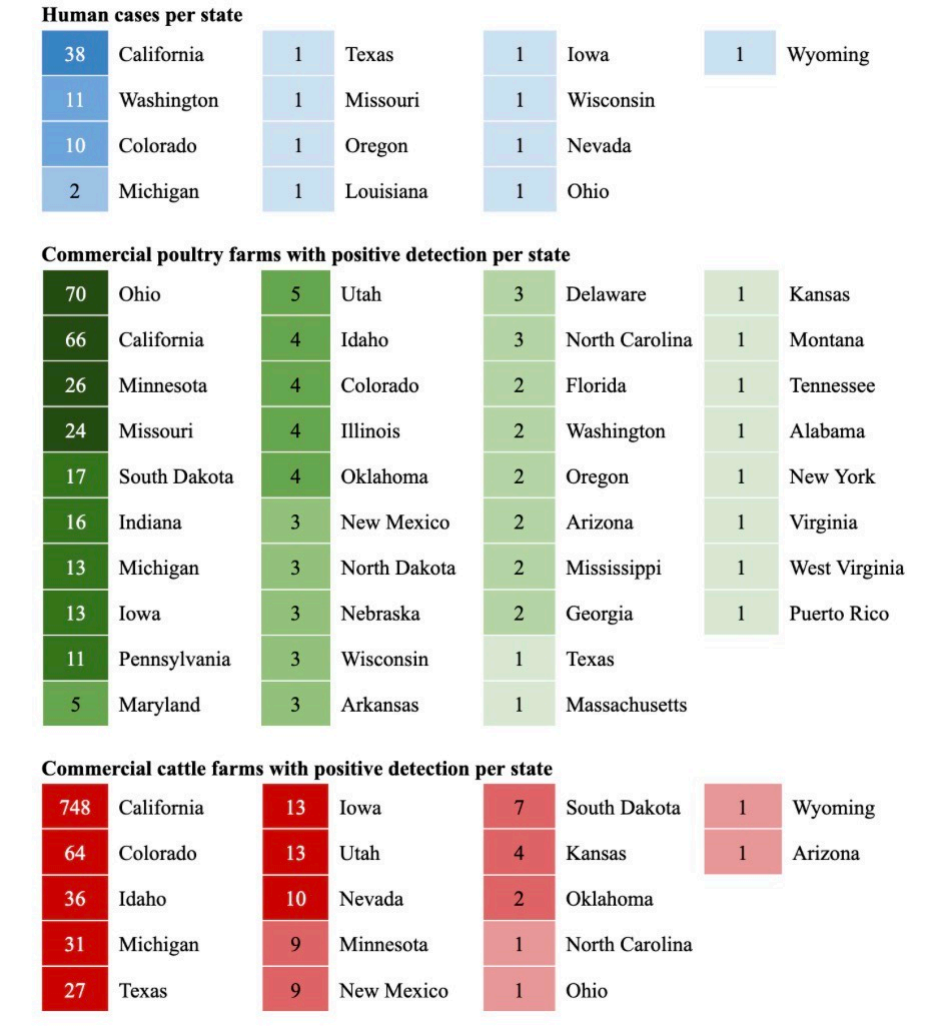
Highly pathogenic avian influenza (HPAI) detections at commercial poultry and cattle farms per state, 2024‐2025.

As of February 28, 2025, the outbreak timeline data consisted of a total of 682 entries across all categories. Sixty entries in the “human” category included information related to HPAI infections in humans (eg, case counts and research updates). As case counts rapidly increased in the fall of 2024, some timeline entries reported multiple newly confirmed human infections on the same day, which is why the number of human infections exceeds the number of entries tagged in the “human” category. However, summing the case reports included in our 60 “human” entries provides the total number of confirmed cases reported in the United States. For animal outbreak data, there were 116 entries in the “birds” category, with the latest timeline entry capturing a total of 325 positive detections at commercial poultry farms across 37 states and 1 US territory (Figure 5). There were 191 entries under the “cattle” category, with the latest timeline entry capturing a total of 977 positive detections at commercial dairy farms across 17 states (Figure 5).

Figure 5 outlines HPAI detections among humans, commercial poultry farms, and commercial cattle farms by state in the United States between February 1, 2024, and February 28, 2025. These charts depict the number of human cases (blue), commercial poultry farms with positive detections (green), and commercial cattle farms with positive detections (red) per state collected through a multimodal data approach. The state sums correspond to the total case reports collected in the timeline, human cases (n=70), commercial poultry farm outbreaks (n=325), and commercial cattle farm outbreaks (n=977). Shading indicates the number of outbreaks per state, where the shading gets darker for states with more detections.

A total of 189 entries in the “wastewater” category displayed wastewater detections across the country. The “wastewater” category displayed results from our observation of positive H5 detections using WastewaterSCAN’s public dashboard in Michigan, Idaho, Texas, Minnesota, Iowa, California, South Dakota, Colorado, Arkansas, Florida, Nevada, Tennessee, Utah, Maine, Delaware, New Jersey, Illinois, Kansas, Connecticut, and Massachusetts as of February 28, 2025 [42].

The timeline also incorporated 31 entries in the “genome” category detailing genomic information about the outbreak and research related to the avian influenza genome. There were 29 entries in the “mammals” category that provided updates on HPAI detections in other mammals besides cattle and humans, such as domestic and feral cats, black rats, pigs, zoo animals (eg, harbor seals), house mice, deer mice, prairie voles, and desert cottontails. Sixty-six entries in the “response” category highlighted response efforts, including policy mandates, and changes to recommendations and guidelines as the outbreak evolved.

### Visualizing Multimodal Data

The outbreak visualization map allows users to explore human cases and animal outbreaks in a single view, supporting a One Health approach to outbreak surveillance [47].

**Figure 6.**
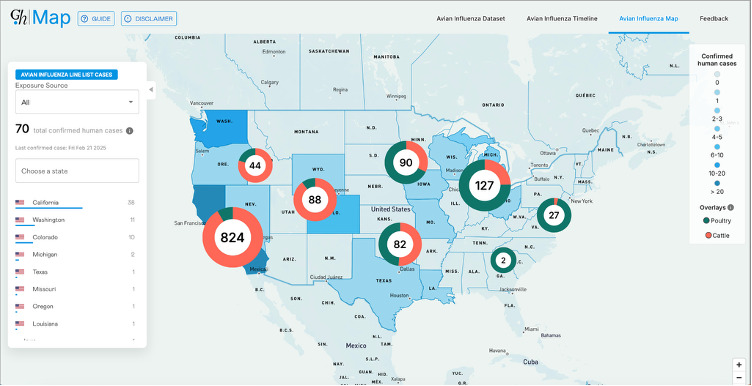
Outbreak visualization map.

Figure 6 shows the spatial distribution of reported cases of HPAI in humans, commercial poultry, and commercial cattle outbreaks as of February 28, 2025 (confirmation dates were used to pinpoint cases in time). During the study period, there were 13 states with confirmed human cases, 17 states with infected commercial cattle, and 37 states plus 1 US territory with infected commercial poultry. Of the confirmed human cases, 41 were the result of exposure to commercial dairy herds (cattle), 24 were the result of exposure to commercial poultry farms and culling operations, 2 resulted from exposure to other animals (ie, other noncommercial birds, including backyard poultry), and 3 had an unknown exposure source. California emerged as a hotspot during the study period, reporting the highest numbers of human cases and commercial cattle outbreaks in the United States. Overall, 54.3% (n=38) of all human cases in the United States were reported in California, including 36 human cases associated with cattle exposure and 2 cases with an unknown exposure source [19].

Figure 6 displays the outbreak visualization map with human and animal surveillance data compiled between February 1, 2024, and February 28, 2025. The map shows the geographic distribution of confirmed human cases and overlays corresponding poultry and cattle outbreak data in a single view to show emerging hotspots. Circle markers indicate regional- or state-level outbreak totals for each species, with the ability to filter and zoom for spatial granularity. A further description of the methods is described in Multimedia Appendix 2.

### Case Study: California

This case study focuses on the temporal progression of HPAI A(H5) detections in public health surveillance data. We examined human health, animal outbreak (commercial cattle and commercial poultry), and wastewater surveillance data reported during the study period from February 1, 2024 through February 28, 2025, filtered to events occurring in California. During the study period, we also recorded response actions, policy changes, and research updates in our outbreak timeline; however, these activities are not the focus of this case study and are acknowledged only for contextual completeness.

In April 2024, WastewaterSCAN developed and evaluated a test targeting the H5 hemagglutinin gene of influenza A for use in wastewater surveillance [44]. Routine H5 testing was implemented at sites nationwide in May 2024, with results publicly reported beginning in June [42,44]. On June 10, 2024, WastewaterSCAN reported the first positive H5 detection at a wastewater treatment site in California [42]. Additional positive wastewater detections were observed in July and September, representing early signals of viral presence in the state before confirmed infections in animals or humans [42], but notably not before the detection of HPAI in wild birds. Although the interpretation of wastewater detections is complex and does not allow attribution to a specific host, wastewater surveillance is recognized as a useful early-warning component of outbreak monitoring [44,57,58].

Eighty-one days after the first positive wastewater detection, on August 30, 2024, the California Department of Food and Agriculture confirmed the state’s first HPAI detection in commercial dairy cattle in Sacramento County within the Central Valley [59].

Before the 2024‐2025 HPAI A(H5) outbreak, the USDA reported HPAI infections among commercial poultry in Fresno County, California, in August 2022 (outbreak reference: Fresno 01). From 2022 to 2024, periods of active detection were interspersed with intervals without reported cases, alongside ongoing efforts by poultry producers to strengthen biosecurity measures to protect flocks and prevent disease spread [8]. The state was subsequently self-declared free of HPAI in poultry on June 28, 2024, following completion of required response actions and surveillance [60]. On September 18, 2024, 100 days after the initial wastewater detection, the USDA confirmed a new HPAI outbreak in commercial poultry in Merced County (outbreak reference: Merced 12), marking the first detection since the state’s HPAI-free declaration and resulting in the loss of that status [60].

On October 3, 2024, the California Department of Public Health announced the state’s first confirmed human cases of HPAI A(H5) in 2 adult dairy farm workers with occupational exposure to infected cattle, occurring 115 days after the first wastewater detection [61]. No epidemiological link between the 2 individuals was identified, and there was no evidence of human-to-human transmission [61].

Positive H5 detections in wastewater continued throughout the remainder of the study period, with an increasing trend observed beginning in October 2024 and peaking between November 2024 and January 2025 [42]. Human infections among individuals with occupational exposure followed a similar temporal pattern, with confirmed cases peaking in October (n=16), November (n=12), and December (n=8) of 2024 [19]. Viral genome sequencing findings supported spillover from dairy cattle as the source of infection among affected farm workers, consistent with observations in other states [62]. However, in most cases, the definitive source of H5 detections in wastewater has not been determined.

**Figure 7.**
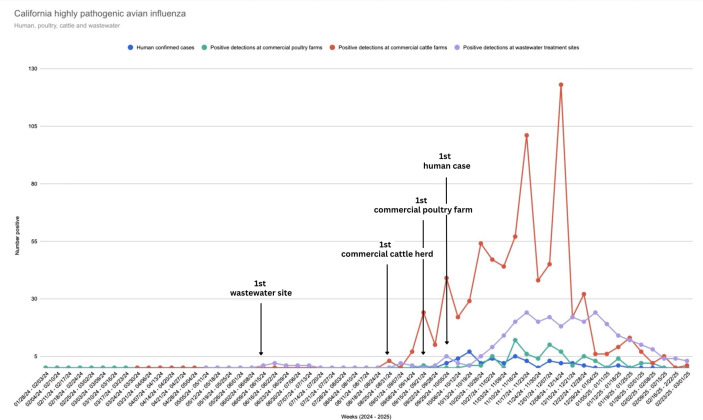
Graph of key data surveillance events in the California outbreak of highly pathogenic avian influenza (HPAI) A(H5) (February 1, 2024, to February 28, 2025).

By the end of the study period, California had reported 748 positive detections among commercial dairy farms and 66 positive detections among commercial poultry farms, illustrating rapid expansion of the outbreak within animal populations [8,41]. These trends are displayed in Figure 7.

Figure 7 displays the time-series analysis used to track HPAI A(H5) activity across multiple data modalities in California. The dataset includes the number of positive human cases, detections at commercial poultry farms, detections at commercial dairy cattle farms, and wastewater treatment site detections. The study period is from February 1, 2024, through February 28, 2025, and dates were matched to CDC epidemiological weeks, ranging from January 28, 2024 (week 5, 2024) through March 1, 2025 (week 9, 2025). Data from WastewaterSCAN were not available before May 7, 2024. Cattle data from the USDA were not available before March 25, 2024. The USDA reports historical poultry outbreak data dating back to the introduction of HPAI in California in 2022. The first positive detection of H5 at a wastewater site in California occurred on June 10, 2024, followed by the first positive detection at a commercial cattle farm on August 30, 2024. There was also a positive detection at a commercial poultry farm on September 18, 2024, the first detection of HPAI in poultry since California was declared HPAI-free in June 2024. The first human cases of HPAI in California were announced on October 3, 2024, 115 days after the first positive detection at a wastewater site.

## Discussion

### Principal Findings

In this study, we examined Global.health’s multimodal data collection, integration, and visualization efforts in response to the 2024‐2025 HPAI A(H5) outbreak using data collected in the United States between February 2024 and February 2025. The data collection efforts resulted in the development of publicly accessible resources to improve contextual understanding and track the outbreak, including the outbreak linelist dataset (human) and outbreak timeline (multimodal data collection: human, animal, wastewater, genomic, research, response, and policy). The data collected were also used to produce an outbreak visualization map (human and animal), which overlaid instances among humans, cattle, and poultry for enhanced data visualization and insight capabilities.

When disparate data points were examined holistically, in real-time—tracking human, animal, and wastewater data together—this approach helped identify emerging hotspots, highlighted outbreak trends, and improved situational awareness. California is examined in this study as an outbreak hotspot that quickly became an epicenter. Wastewater was an early indication of the virus in the state, with positive A(H5) detection of unknown source origin in June 2024 [42].

In the context of the 2024‐2025 HPAI A(H5) outbreak, the time between initial detection in wastewater and cases in animals and humans highlights an important window of opportunity for early response. Monitoring of wastewater data coupled with trend analysis, taking into account events such as annual bird migration, can provide insight into changes in trends that can point to emerging outbreaks. Monitoring of these trends within the context of contributing sources is important for the identification of zoonotic diseases with pandemic potential, such as HPAI [63]. Further, a coordinated and collaborative One Health approach to better understand the source could guide public health actions to hone methods and protocols for future respiratory illness seasons or outbreaks (eg, a positive H5 detection in wastewater may prompt further monitoring or testing in animals, milk, or humans; or identify the need for deployment of wastewater testing to high-priority locations to focus on areas with a higher risk of disease transmission) [58].

This outbreak demonstrated key challenges and complexities with wastewater surveillance of HPAI noted in prior literature: an inability to distinguish between human and animal sources [64]; nonhuman and noncattle animal inputs, including wild and migratory birds [64,65]; industrial and agricultural inputs into wastewater [63]; stormwater contributions [64-66]; timely data integration to address knowledge gaps [58]; unknown thresholds for sensitivity in testing and positive detection [58]; current testing is not specific for H5N1 and may also detect low-pathogenic H5 influenza viruses [67]; and differences in laboratory approaches and reporting practices may complicate interpretation [58].

Although waste processing from industrial dairy farms in the United States is highly regulated and, as a result, most farms have internal systems such that waste is not designed to be dumped into municipal sewage systems, research has demonstrated that animal inputs have been responsible for some wastewater detections [44,63,68]. Dairy processing discharge into sewage systems has been studied with evidence to support the hypothesis of dairy industry contributions and the likelihood of multiple sources feeding into wastewater treatment plants [63]. Retrospective testing in other affected areas (Texas and North Carolina) has also shown that H5 was detectable in wastewater before the state identified H5-infected cattle, providing early warning for outbreaks that may contribute to the sewershed [63].

Further, the A(H5) virus is enzootic in wild birds, and animal sources should be considered when interpreting influenza subtype detections in wastewater [64]. Wild birds can be asymptomatic carriers of HPAI [6,69], and the movement and congregation of birds during migration, among all 4 US migratory flyways [70], and on a broader intercontinental scale, where birds may live or stop in different habitats [71], have been shown to be important factors in the geographic spread and ongoing circulation and phylogenetic changes of avian influenza viruses [52,72-77]. California is in the Pacific Flyway, a major migratory route for more than a billion visiting birds each year [78], creating risk for spillover events and point-source transmissions [72], where previous studies have aimed to model high-risk areas in the state [79,80]. The USDA conducts wild bird surveillance for avian influenza viruses of concern and reports positive detections of HPAI in wild birds and poultry in California before and during our study period [8,69]. However, wild bird surveillance data were not collected as part of this study. Wild bird detections are discussed in this context as a potential source contributing to the sewershed and subsequent positive detection in wastewater surveillance data [65]. Further research is needed to assess the geographic representativeness of site detections in California, including associations with migratory bird activity, seasonality of positive detections [81], historical avian influenza detections, and proximity to commercial agriculture.

While the interpretation of the virus in wastewater is complex, it is recognized as an important tool in the surveillance of emerging pathogens and has the potential to improve our understanding of outbreak dynamics and strengthen ongoing avian influenza surveillance efforts, with implications for animal and human health [44,64]. Wastewater data can complement traditional epidemiological data and existing health-monitoring systems [58,82], as demonstrated by Global.health’s multimodal data approach to comprehensive disease surveillance.

Future work should focus on determining the origin of H5 in US municipal wastewater systems and include efforts to sequence viral genome fragments present in wastewater samples. Further, there is a critical need for clear communication to explain the meaning and interpretation of detection for influenza A virus and subtypes in wastewater.

The study demonstrates how the compilation of open-source multimodal data can provide early situational awareness and a more complete view of emerging zoonotic outbreaks. Open-source tools that are able to adapt and scale in real time as an outbreak evolves and presents new challenges, integrating new data sources for enhanced surveillance, are in line with broader calls to modernize pandemic intelligence by leveraging cross-sectoral and nontraditional data streams [83]. Global.health was able to quickly adapt the outbreak schema for the standardized collection of key information pertinent to this pathogen. In this study, we highlighted observations gained from the integration of data from official and nonofficial media sources. Our open-source format supports a transparent approach to information sharing, especially in the early days when there may be delays in official reporting, siloed and fragmented data, and data vacuums. This integration and methodology presents an innovative, valuable, and repeatable approach for open-source public health surveillance for zoonotic or other emerging or reemerging pathogens.

### Limitations

Global.health strives to ensure that the data collated and shared on our platform are timely and accurate. In addition to the limitations associated with interpretation discussed in the Discussion section, (eg, the source of H5 signals in wastewater), there are important limitations associated with the data collected by Global.health during the 2024‐2025 HPAI A(H5) outbreak. As part of quality assurance, data requires continuous reconciliation and validation across local, state, national, and international datasets to ensure alignment and to track changes. All Global.health data collection relies on publicly available data that are deidentified. Further research is needed to realize the full potential of open-source, multimodal data integration to support outbreak response and the impact it can have on public health as a whole.

Curators faced many challenges while building an emerging infectious disease dataset in real time, including delays in reporting for human cases, animal outbreaks, and wastewater detections; delays in confirmatory testing; limited data standardization within and across government organizations; lack of precision of virus nomenclature and terminology (eg, avian influenza, bird flu, AI, HPAI, HPAI A(H5), HPAI A(H5N1), HPAIV, H5, H5Nx, H5N1, and H5-type being used inconsistently and interchangeably in media sources and scientific literature); presentation of aggregated case data; limited case metadata; and retroactive removal and addition of impacted herds, flocks, and wastewater sites. USDA reports confirmed outbreak events by date and location using outbreak reference numbers (also referred to as special IDs). Global.health conducted regular data reconciliation efforts to mitigate retroactive, unannounced, and unexplained changes and data discrepancies between Global.health and our primary sources (WastewaterSCAN and USDA). The average look-back period was 1 week for Global.health to check and update data and was limited by time and available resources. USDA published the total number of cattle outbreaks on their website, and we were able to align counts on a daily basis. However, cumulative numbers were not available from USDA (Poultry) or WastewaterSCAN. Therefore, publicly available data from these sources may differ from the results of this study. These challenges reflect longstanding gaps in US surveillance systems; however, advanced tools and approaches create opportunities to integrate data not traditionally accessible to public health [84]. Innovative methods, such as artificial intelligence and automated quality control tools, may also help enhance data quality in real time [85,86]. To ensure data transparency, all outbreak resources are publicly accessible and point back to the original sources of information.

Primary sources for multimodal data collection are listed for this research. Additional relevant sources may be available, but were not included in this study.

### Conclusions

As outbreaks and the public health landscape evolve, so too must surveillance methods; how data are gathered, analyzed, and visualized matters. Further, the 2024‐2025 HPAI A(H5) outbreak in the United States again highlighted the importance of a One Health approach as the pandemic potential of zoonotic outbreaks grows [87]. Focusing on the interplay of humans, animals, and the shared environment will assist researchers, public health teams, and communities in better understanding the origins, evolution, and scope of emerging zoonotic events. Our data collection efforts and research showed both the value and the challenges of integrating disparate sources to modernize surveillance methods and create public resources that enhance early situational awareness. The potential for wastewater surveillance, and other emerging data modalities, to provide an early indication of disease in a community calls for increased research into how to interpret positive detections in the context of multimodal datasets. Accurate, accessible, real-time data are the cornerstone of prudent outbreak response. Early, open access to data and visualization tools, such as those put forward by Global.health, can demonstrate value in a public health response approach that is adaptable and resilient in the face of emerging threats.

## Supplementary material

10.2196/86209 Multimedia Appendix 1 Schema

10.2196/86209 Multimedia Appendix 2 Methods for visualizing multimodal data.

10.2196/86209 Checklist 1 RECORD checklist.

## Abbreviations

CDC: Centers for Disease Control and Prevention
HPAI: highly pathogenic avian influenza
NWSS: National Wastewater Surveillance System
STROBE: Strengthening the Reporting of Observational Studies in Epidemiology
USDA: United States Department of Agriculture
WOAH: World Organization for Animal Health
