# Supplementary material for: Multimodal Data Approaches for Examining the 2024-2025 Highly Pathogenic Avian Influenza Outbreak in the United States: Descriptive Study
Source: JMIR Public Health Surveill. 2026 Jun 22;12:e86209. doi: 10.2196/86209 (PMC13286079; doi:10.2196/86209)
Supplement: Multimedia Appendix 1 [file publichealth-v12-e86209-s001.pdf]

Table 2: Schema

Variable Name	Definition	Value
ID	Unique identification number of the case	*Number*. Starting with 1 and incrementing for each new case.
Pathogen_name	Pathogen of case	*String*. Constant pre-filled value: Influenza A
Pathogen_subtype1	Pathogen subtype for Influenza A Hx. Hemagglutinin protein type	*String*. Hx
Pathogen_subtype2	Pathogen subtype for Influenza A, further classification HxNy. Hemagglutinin protein and neuraminidase protein types	*String*. HxNy
Case_status	Status of the case	*String*. Must be one of [“confirmed”, “probable”, “suspected”, “discarded”, “omit_error”]
Location_Admin0	Admin 0 location [country]	*String*, countries (states or territories) that have ISO 3166-1 codes, USA perspective is used for disputed areas
Location_Admin1	Admin 1 location [state / province]	*String*, first-level administrative areas that have ISO 3166-2 (ISO 3166-1 alpha-2) codes, usually states or provinces
Location_Admin2	Admin 2 location [county / district]	*String*, second-level administrative areas, like counties, districts, or cities
Location_Admin3	Admin 3 location [city]	*String*, third-level administrative areas, like townships, towns, municipalities or villages
Age	Age of individual	*String*. Specified as range, open-ended (<n, >n) or as a range delimited by a hyphen following 5 year increments (m-n). Open-ended values for ‘adult’ is >=18 and ‘child’ is <18.
Gender	Sex at birth of an individual	*String*. Must be one of [“male”, “female”, “other”]
Occupation	Individual’s occupation	*String*. Free response entry
Symptoms	Comma separated list of symptoms as described by source	*String*. Individual symptoms separated by comma. List of individual symptoms recorded as of February 28, 2025 is presented in Table 5.
Date_onset	Date of onset of symptoms	*String, format: iso8601date*
Date_confirmation	Date case was confirmed	*String, format: iso8601date*
Pre_existing_condition	If the individual has any pre-existing conditions)	*String*. Must be one of [“Y”, “N”]
Hospitalised	Whether individual was hospitalized	*String*. Must be one of [“Y”, “N”]
Date_hospitalisation	Date individual was hospitalized	*String, format: date*
Intensive_care	Whether individual admitted to an intensive care unit or high dependency unit at hospital	*String*. Must be one of [“Y”, “N”]
Isolated	Whether individual was isolated at home or in hospital	*String*. Must be one of [“Y”, “N”]
Date_isolation	Date individual entered isolation	*String, format: iso8601date*

Treatment_antiviral	Has the individual received antiviral treatment	*String, Must be one of* [“Y”, “N”]
Treatment_antiviral_name	Name of antiviral drug	*String*.
Vaccination	Has the individual received a dose of vaccine	*String*. Must be one of [“Y”,”N”]
Vaccine_name	Name of the first vaccine	*String*.
Vaccine_date	Date of first vaccination	*String, format: iso8601date*
Outcome	Outcome of disease, recovered includes inactive case counts.	*String*. Must be one of [“recovered”, “death” , “ongoing post-acute condition”]. “Recovered” includes ‘recovering’ outcome status, as described by Source
Date_death	Date individual died	*String, format: iso8601date*
Contact_with_case	Has the individual had contact with a confirmed or suspected case	*String*. Must be one of [“Y”, “N”]
Contact_ID	If specified, the ID of the confirmed or suspected contact	*String*.
Contact_setting	Setting where contact occurred	*String*. Must be one of [“HOUSE”, “WORK”, “SCHOOL”, “HEALTH”, “PARTY”, “BAR”, “LARGE”, “LARGECONTACT”, “OTHER”, “UNK”]
Contact_animal	Whether the individual has known contact with animals	*String*. Must be one of [“COMMERCIAL”, “BACKYARD”, “PET”, “PETRODENTS”, “WILD”, “WILDRODENTS”, “OTHER”]
Contact_animal_species	Free text describing animal species with known contact	*String*.
Transmission	Most likely mode of transmission	*String*. Must be one of [“ANIMAL”, “HAI”, “LAB”, “MTCT”, “OTHER”, “FOMITE”, “PTP”, “SEX”, “TRANSFU”, “UNK”]
Travel_history	Whether an individual has travel history, domestic and/or international	*String*. Must be one of [“Y”, “N”]
Travel_history_entry	Date when individual entered the country	*String, format: iso8601date*
Travel_history_start	Date when individual began travel	*String, format: iso8601date*
Travel_history_location	Location of travel obtained from geocoding API such as Mapbox	*String*, free-text field for location information*Object (GeoJSON)*
Genomic_Sequence	Sequence name and accession number(s) uploaded to public database	*String*.
Genomics_Clade	Which clade the viral strain belongs to	*String*.
Genomics_Genotype	Which genotype the viral strain belongs to	*String*.
Confirmation_method	Test used to perform diagnosis	*String*.
Source	URL of news story or government source where this case was confirmed	*String, format: url*
Source_Date report	Date of report for Source (I)	*String, format: iso8601date*
Source_Government	Indicate whether Source (I) is an official government source	*String*. Must be one of [“Y”, “N”]

Source_II	URL of news story or government source where this case was confirmed	*String, format: url*
Source_III	URL of news story or government source where this case was confirmed	*String, format: url*
Source_IV	URL of news story or government source where this case was confirmed	*String, format: url.*
Source_V	URL of news story or government source where this case was confirmed	*String, format: url.*
Source_VI	URL of news story or government source where this case was confirmed	*String, format: url.*
Source_VII	URL of news story or government source where this case was confirmed	*String, format: url.*
Source_VIII	URL of news story or government source where this case was confirmed	*String, format: url.*
Source_IX	URL of news story or government source where this case was confirmed	*String, format: url.* Additional sources may be added.
Location_Comment*	Location detail for case	*String*. For internal use only.
Contact_comment*	Free text describing any additional contact information	*String*. For internal use only
Travel_history_comment*	Travel history detail for case	*String*. For internal use only
Confirmation_comment*	Testing or confirmation detail for case	*String*. For internal use only
Date_entry*	Date case was entered into linelist	*String, format: iso8601date*
Date_last_modified*	Last date when case was modified in linelist	*String, format: iso8601date.*
Curator_initials*	Curator initials of those who contributed to curating case	*String*. For internal use only
Verified*	Whether case has been verified by second curator	*String*. Must be one of ["Y", "N"]. For internal use only
Verified_by*	Initials of verifier	*String*. For internal use only
Curator Comment*	Curator notes about case	*String*. For internal use only

Table 2 outlines all schema variables used by the Global.health curators during the 2024 - 2025 outbreak response to highly pathogenic avian influenza in the United States.
* Indicates that this variable was private and could only be viewed by the curation team
