# Supplementary material for: Multimodal Data Approaches for Examining the 2024-2025 Highly Pathogenic Avian Influenza Outbreak in the United States: Descriptive Study
Source: JMIR Public Health Surveill. 2026 Jun 22;12:e86209. doi: 10.2196/86209 (PMC13286079; doi:10.2196/86209)
Supplement: Multimedia Appendix 2 [file publichealth-v12-e86209-s002.docx]

In order for data to be properly displayed on the map, it needs to contain identifying information about affected locations. Global.health utilizes admin0 to admin3 administrative resolutions (those correspond to: *admin0* - country - ISO_3166-1, *admin1* - states or provinces - ISO_3166-2, *admin2* - counties, districts, or cities, *admin3* - townships, towns, municipalities or villages). For this event, animal outbreak data are available and displayed at admin2 (county), and human cases are displayed at admin1 (state). The location data identifiers are then parsed to the mapbox compatible format using their proprietary data format [[88]](https://www.zotero.org/google-docs/?6Z37Io). The parsing process is scheduled to take place daily at 1AM UTC, and we make sure to save parsed data snapshots everyday to be able to access it if necessary. Different colors are used to depict the different species affected (human: blue, cattle: orange, poultry: green). A color gradient and scale are applied to human cases at the state level (admin1). The date range captured on the map spans from March 7, 2024 to February 28, 2025. USDA identified a poultry outbreak, classified under the “WOAH Poultry” category, in Essex County, Massachusetts confirmed on March 7, 2024. No data for confirmed human, cattle, or poultry cases were identified prior to this date inside the study period following our inclusion criteria.

The total number of human HPAI cases across all affected US states is listed on the left side menu bar of the map. Human HPAI cases can be filtered by Exposure Source, currently containing five possible values: All, Cattle, Poultry, Other Animal Exposure, and Exposure Source Unknown. Additional exposure sources are added automatically as they appear in the Outbreak Linelist. State-level breakdowns can be accessed by clicking on the state area on the choropleth map or selecting it from the side menu.

Two overlays for commercial cattle and poultry outbreaks are available to select on the right side menu of the map. Each overlay represents data in *admin2* (county) administrative level. Those overlays can be toggled on and off using a side menu. Overlays are represented as markers that display the number of outbreaks and a doughnut chart showing the cattle-to-poultry ratio. Overlays form clusters that are grouped together according to the current map zoom level, clicking on the cluster zooms in the map view to the level where clusters can be split into smaller clusters and eventually individual affected areas. Hovering over animal overlay markers triggers a popup that contains additional information (commercial poultry and cattle outbreak count, affected state(s) and county(s), and last recorded outbreak). Map data is refreshed daily. The map interface also provides quick links to the Outbreak Linelist, Outbreak Timeline, and provides users with a map guide and disclaimer to help them understand and interact with this tool.
