# Supplementary material for: Multimodal Data Approaches for Examining the 2024-2025 Highly Pathogenic Avian Influenza Outbreak in the United States: Descriptive Study
Source: JMIR Public Health Surveill. 2026 Jun 22;12:e86209. doi: 10.2196/86209 (PMC13286079; doi:10.2196/86209)
Supplement: Checklist 1 [file publichealth-v12-e86209-s003.docx]

**The RECORD statement – checklist of items, extended from the STROBE statement, that should be reported in observational studies using routinely collected health data.**

|  | **Item**  **No.** | **STROBE items** | **Location in**  **manuscript where items are reported** | **RECORD items** | **Location in**  **manuscript**  **where items are reported** |
| --- | --- | --- | --- | --- | --- |
| **Title and abstract** | | | | | |
|  | 1 | (a) Indicate the study’s design with a commonly used term in the title or the abstract (b)  Provide in the abstract an  informative and balanced  summary of what was done and what was found | 1. This is a descriptive observational surveillance study. “Descriptive study” design is included in the title. (Title; Abstract, pp. 1-2) 2. The abstract provides an informative and balanced summary of the study objectives, methods, and key findings. (Abstract, p. 2-3) | RECORD 1.1: The type of data used should be specified in the title or abstract. When possible, the name of the databases used should be included.  RECORD 1.2: If applicable, the geographic region and timeframe within which the study took place should be reported in the title or abstract.  RECORD 1.3: If linkage between databases was conducted for the study, this should be clearly stated in the title or abstract. | 1.1) The abstract specifies the use of multimodal, publicly available data sources, including human case data, animal outbreak reports, wastewater surveillance, genomic data, and policy, research, and response information. Primary data sources are described. (Abstract, ‘*Methods*’, p. 2)  1.2) The methods section within the abstract includes our study period (February 1, 2024-February 28, 2025) and location (United States). (Abstract, ‘*Methods*’, p. 2)  1.3) Not applicable. No data linkage was performed. |
| **Introduction** | | | | | |
| Background  rationale | 2 | Explain the scientific  background and rationale for the investigation being reported | The Introduction describes a public health threat - the emergence of HPAI (H5N1) as a multispecies outbreak in the US - and the resulting challenges posed by fragmented, siloed data systems. The Global.health platform and the rationale for applying an integrated, multimodal, One Health surveillance approach for enhanced, early situational awareness is explained. (Introduction, ‘*Background*’ p. 4) |  |  |
| Objectives | 3 | State specific objectives,  including any prespecified hypotheses | The study objectives are clearly stated: to describe observations from the application of multimodal data collection, integration, and visualization for real-time outbreak surveillance. No hypotheses were prespecified, consistent with the descriptive study design. (Introduction, ‘*Objectives*’, pp. 4-5) |  |  |
| **Methods** | | | | | |
| Study Design | 4 | Present key elements of study design early in the paper | Key elements of the descriptive observational surveillance study design are presented in the Methods, including multimodal data collection, curation, and visualization through outbreak line-lists, timelines, and maps. (Methods, pp. 5-9) |  |  |
| Setting | 5 | Describe the setting, locations, and relevant dates, including periods of recruitment, exposure, follow-up, and data collection | The setting (United States) and study period (February 1, 2024-February 28, 2025) are described in Methods. The study used secondary, de-identified data from publicly available sources for public health surveillance; therefore, there was no participant recruitment, direct exposure assessment, or follow-up at the individual level. (Methods, pp. 6-7) |  |  |

| Participants | 6 | *(a) Cohort study*- Give the eligibility criteria, and the  sources and methods of selection of participants. Describe  methods of follow-up  *Case-control study*- Give the eligibility criteria, and the  sources and methods of case ascertainment and control  selection. Give the rationale for the choice of cases and controls *Cross-sectional study*- Give the eligibility criteria, and the  sources and methods of selection of participants  *(b) Cohort study*- For matched studies, give matching criteria and number of exposed and unexposed  *Case-control study*- For  matched studies, give matching criteria and the number of  controls per case | Not applicable. This is a descriptive observational surveillance study using secondary, publicly available, de-identified data and does not involve recruitment of participants. Inclusion criteria for reported cases and outbreak events, along with primary data sources and case definitions, are described in the Methods, if applicable. (Methods, pp. 5-9) | RECORD 6.1: The methods of study population selection (such as codes or algorithms used to identify subjects) should be listed in detail. If this is not possible, an explanation should be provided.  RECORD 6.2: Any validation studies of the codes or algorithms used to select the population should be  referenced. If validation was conducted for this study and not published elsewhere, detailed methods and results should be provided.  RECORD 6.3: If the study involved linkage of databases, consider use of a flow diagram or other graphical display to demonstrate the data linkage process, including the number of individuals with linked data at each stage. | 6.1) Study inclusion for reported cases and outbreak events was based on publicly available, de-identified data. No codes or algorithms were used to identify subjects; inclusion criteria and case definitions are described in the Methods, if applicable. (Methods, pp. 5-9)  6.2) Not applicable. No codes or algorithms were used to select the study population; therefore, no validation studies were required.  6.3) Not applicable. No data linkage was performed, and no linkage flow diagram was required. |
| --- | --- | --- | --- | --- | --- |
| Variables | 7 | Clearly define all outcomes, exposures, predictors, potential confounders, and effect  modifiers. Give diagnostic  criteria, if applicable. | Primary outcomes are defined using source-specific reporting criteria for confirmed human cases, animal outbreaks, and positive wastewater detections (e.g., confirmed human case status and exposure source follows CDC guidance). Given the descriptive study design, no predictors, potential confounders, or effect modifiers were specified or analyzed. (Methods, pp. 6-9) | RECORD 7.1: A complete list of codes and algorithms used to classify exposures, outcomes, confounders, and effect modifiers should be provided. If these cannot be reported, an  explanation should be provided. | 7.1) Not applicable. No codes or algorithms were used to classify variables. |
| Data sources/  measurement | 8 | For each variable of interest, give sources of data and details of methods of assessment  (measurement).  Describe comparability of  assessment methods if there is more than one group | For each multimodal data stream, sources and methods of data collection are described. Timeline categories and their corresponding data sources are defined. (Methods, pp. 6-9 ) |  |  |

| Bias | 9 | Describe any efforts to address potential sources of bias | Potential sources of bias related to reporting delays and data completeness are acknowledged. Regular data review was conducted to reduce inaccuracies. (Discussion, ‘*Limitations*’, pp. 17-18) |  |  |
| --- | --- | --- | --- | --- | --- |
| Study size | 10 | Explain how the study size was arrived at | Study size reflects the total number of confirmed human cases, animal outbreak events, and positive wastewater detections available during the study period. (Results, pp. 9-16) |  |  |
| Quantitative variables | 11 | Explain how quantitative  variables were handled in the analyses. If applicable, describe which groupings were chosen, and why | This study reports descriptive counts and does not include statistical analyses. Quantitative variables were presented as reported by source (e.g., counts over time or by location) and were not transformed or modeled. |  |  |
| Statistical methods | 12 | (a) Describe all statistical  methods, including those used to control for confounding  (b) Describe any methods used to examine subgroups and interactions  (c) Explain how missing data were addressed  (d) *Cohort study*- If applicable, explain how loss to follow-up was addressed  *Case-control study*- If  applicable, explain how  matching of cases and controls was addressed  *Cross-sectional study*- If  applicable, describe analytical methods taking account of sampling strategy  (e) Describe any sensitivity analyses | Not applicable. The study reports descriptive counts and summaries only; no statistical modeling or inferential analyses were performed. |  |  |
| Data access and cleaning methods |  | .. |  | RECORD 12.1: Authors should describe the extent to which the investigators had access to the database population used to create the study population. | Curators accessed the same publicly available data directly from source websites. No restricted databases were used. (Methods, pp. 6-9) |

|  |  |  |  | RECORD 12.2: Authors should provide information on the data cleaning methods used in the study. | Data cleaning included standardization of variable names, removal of duplicate entries, and reconciliation of conflicting reports across sources. No imputation of missing data was performed. (Methods, pp. 6-9) |
| --- | --- | --- | --- | --- | --- |
| Linkage |  | .. |  | RECORD 12.3: State whether the study included person-level,  institutional-level, or other data linkage across two or more databases. The methods of linkage and methods of linkage quality evaluation should be provided. | 12.3) Not applicable. No data linkage was performed. |
| **Results** | | | | | |
| Participants | 13 | (a) Report the numbers of  individuals at each stage of the study (*e.g.*, numbers potentially eligible, examined for eligibility, confirmed eligible, included in the study, completing follow-up, and analysed)  (b) Give reasons for non-  participation at each stage.  (c) Consider use of a flow  diagram | Individual participant flow is not applicable. Results summarize confirmed human cases, animal outbreak events, and positive wastewater detections, derived from publicly available sources. (Results, pp. 9-16) | RECORD 13.1: Describe in detail the selection of the persons included in the study (*i.e.,* study population selection) including filtering based on data quality, data availability and linkage. The selection of included persons can be described in the text and/or by means of the study flow diagram. | Included cases were publicly reported (e.g. confirmed human HPAI cases meeting source-defined criteria for our setting and study period). Data availability varied across sources due to public reporting practices; no data linkage was performed. (Results, pp. 9-10) |
| Descriptive data | 14 | (a) Give characteristics of study participants (*e.g.*, demographic, clinical, social) and information on exposures and potential  confounders  (b) Indicate the number of participants with missing data for each variable of interest (c) *Cohort study*- summarise follow-up time (*e.g.*, average and total amount) | 1. Descriptive characteristics of confirmed human cases (e.g., demographics, exposure source, pathogen subtype) are presented in the Results. Individual follow-up time is not applicable. (Results, pp. 9-10) 2. Data completeness was not assessed for all variables; variable-level data availability for symptomatology is reported in Supplementary Table 1. (Table 1, p. 10) |  |  |
| Outcome data | 15 | *Cohort study*- Report numbers of outcome events or summary measures over time  *Case-control study*- Report numbers in each exposure | Numbers of confirmed human cases, reported animal outbreaks are summarized over the study period. (Results, pp. 9-16) |  |  |

|  |  | category, or summary measures of exposure  *Cross-sectional study*- Report numbers of outcome events or summary measures |  |  |  |
| --- | --- | --- | --- | --- | --- |
| Main results | 16 | (a) Give unadjusted estimates and, if applicable, confounder- adjusted estimates and their precision (e.g., 95% confidence interval). Make clear which confounders were adjusted for and why they were included (b) Report category boundaries when continuous variables were categorized  (c) If relevant, consider  translating estimates of relative risk into absolute risk for a meaningful time period | Not applicable. No effect estimates or adjusted analyses were conducted. |  |  |
| Other analyses | 17 | Report other analyses done— e.g., analyses of subgroups and interactions, and sensitivity analyses | Not applicable. No subgroup, interaction, or sensitivity analyses were performed. |  |  |
| **Discussion** | | | | | |
| Key results | 18 | Summarise key results with reference to study objectives | Key findings are summarized in relation to the study objectives, demonstrating how multimodal, publicly available data were used within a One Health framework to characterize the 2024–2025 US HPAI outbreak and support early situational awareness via the Global.health platform. (Discussion, Principal Findings, pp. 16-17) |  |  |
| Limitations | 19 | Discuss limitations of the study, taking into account sources of potential bias or imprecision. Discuss both direction and magnitude of any potential bias | Limitations of the study are described in the Discussion, including potential sources of bias or imprecision. The direction and magnitude of these biases were not explicitly assessed due to the descriptive, surveillance-based design. (Discussion, ‘Limitations’, pp. 17-18) | RECORD 19.1: Discuss the  implications of using data that were not created or collected to answer the specific research question(s). Include discussion of misclassification bias, unmeasured confounding, missing data, and changing eligibility over time, as they pertain to the study being reported. | 19.1) The Discussion addresses the limitations of using secondary, de-identified, publicly available data that were not created for our specific research objectives. (Discussion, ‘Limitations’, pp. 17-18) |
| Interpretation | 20 | Give a cautious overall  interpretation of results  considering objectives, limitations, multiplicity of analyses, results from similar studies, and other relevant evidence | Findings are interpreted cautiously in light of study objectives, limitations, and existing evidence, including challenges in interpreting wastewater surveillance signals. (Discussion, pp. 16-17) |  |  |

| Generalisability | 21 | Discuss the generalisability (external validity) of the study results | The Results demonstrate the value of a multimodal One Health surveillance approach to support early situational awareness. The study’s findings are generalisable, as Global.health’s open source platform is flexible, scalable, and adaptable to other zoonotic and emerging infectious disease contexts, enabling real-time, transparent integration of diverse data sources to create outbreak resources. (Results, pp.16-18) |  |  |
| --- | --- | --- | --- | --- | --- |
| **Other Information** | | | | | |
| Funding | 22 | Give the source of funding and the role of the funders for the present study and, if applicable, for the original study on which the present article is based | Sources of funding and the role of funders are disclosed (p. 19) |  |  |
| Accessibility of protocol, raw  data, and  programming  code |  | .. |  | RECORD 22.1: Authors should provide information on how to access any supplemental information such as the study protocol, raw data, or programming code. | 22.1) Information on access to supplemental materials, curated datasets, and outbreak resources is provided. (‘Data Availability’, p. 18) |

*Reference: Benchimol EI, Smeeth L, Guttmann A, Harron K, Moher D, Petersen I, Sørensen HT, von Elm E, Langan SM, the RECORD Working Committee. The REporting of studies Conducted using Observational Routinely-collected health Data (RECORD) Statement. *PLoS Medicine* 2015; in press.

*Checklist is protected under Creative Commons Attribution (CC BY) license.
